# Dasatinib and Quercetin alleviate type 2 diabetic osteoporosis by regulating serum metabolite and gut microbiome

**DOI:** 10.3389/fmicb.2025.1631082

**Published:** 2025-09-03

**Authors:** Junzheng Yang, Hua Zhang, Zhuoxu Gu, Guanghui Zhou, Guihong Liang, Lingfeng Zeng, Jinlong Zhao, Weiyi Yang, Jun Liu, Jianke Pan

**Affiliations:** ^1^Fifth School of Clinical Medicine, Guangzhou University of Chinese Medicine, Guangzhou, Guangdong, China; ^2^Guangdong Provincial Second Hospital of Traditional Chinese Medicine (Guangdong Provincial Engineering Technology Research Institute of Traditional Chinese Medicine), Guangzhou, Guangdong, China; ^3^Guangdong Provincial Key Laboratory of Research and Development in Traditional Chinese Medicine, Guangzhou, Guangdong, China; ^4^Bone and Joint Research Team of Degeneration and Injury, Guangdong Provincial Academy of Chinese Medical Sciences, Guangzhou, China; ^5^First School of Clinical Medicine, Guangzhou University of Chinese Medicine, Guangzhou, Guangdong, China; ^6^The Second Affiliated Hospital of Guangzhou University of Chinese Medicine (Guangdong Provincial Hospital of Chinese Medicine), Guangzhou, China; ^7^Guangdong Provincial Key Laboratory of Chinese Medicine for Prevention and Treatment of Refractory Chronic Diseases, Guangzhou, China

**Keywords:** senolytics, bone metabolic diseases, gut microbiota, metabolomics, type 2 diabete mellitus

## Abstract

Type 2 diabetic osteoporosis (T2DOP) is a complex metabolic bone disorder characterized by reduced bone density and increased risk of osteoporosis in patients with type 2 diabetes mellitus. The etiology of T2DOP is multifactorial, involving hyperglycemia, insulin resistance, and gut microbiota dysbiosis. Current management strategies for T2DOP typically involve a comprehensive approach, including strict glycemic control, vitamin D and calcium supplementation, anti-osteoporotic medications, increased physical activity, and gut microbiota modulation. This study aimed to investigate the therapeutic potential of the combination of Dasatinib and Quercetin (D + Q), known as senolytics, in treating T2DOP. To elucidate the underlying mechanisms, a well-characterized T2DOP mouse model was established. Bone mass was evaluated using micro-computed tomography and histological staining techniques. Subsequently, the impact of D + Q treatment on gut microbiota composition and complex serum metabolite profiles was comprehensively examined. The results demonstrated that D + Q reshaped gut microbiota, resulting in increased short-chain fatty acid producers (*Lachnospiraceae* and *Bacteroides*) and decreased proinflammatory bacteria (*Mucispirillum*), which were associated with the therapeutic effects in bone-fat balance. Additionally, D + Q treatment enhanced amino acid and short-chain fatty acid metabolism while simultaneously reducing cholesterol and triglyceride levels.

## Introduction

Type 2 diabetic osteoporosis (T2DOP) is characterized by secondary bone loss induced by type 2 diabetes mellitus (T2DM; Ebeling et al., 2022). The relationship between T2DM and decreased bone mineral density (BMD) remains a subject of ongoing debate. A survey based on data from the National Health and Nutrition Examination Survey revealed that the prevalence of osteoporosis (OP) was significantly higher among individuals with T2DM (Xu and Wu, 2021; Ma et al., 2012). Moreover, accumulating evidence indicates that patients with T2DM exhibit an increased risk of fractures even if there is no significant decrease in BMD. These findings indicate that metabolic dysregulation in T2DM exacerbates skeletal fragility (Hamann et al., 2012; Valderrábano and Linares, 2018).

T2DM was associated with an increased burden of senescent cells. Concurrently, cellular senescence contributed to T2DM development (Palmer et al., 2015). The hallmark features of T2DOP, including decreased autophagy, elevated levels of advanced glycation end products, and bioactive lipids accumulation, are indicators of the aging process (Farr et al., 2017; Xu et al., 2015). Currently, 46 potential senolytic compounds have been identified. A combination of Dasatinib and Quercetin (D + Q) is one of the most studied senolytics used to improve common complications of T2DM, including liver fat accumulation and consequent cirrhosis (Kirkland and Tchkonia, 2020). The effects of D + Q on T2DOP remain unclear.

The human gut harbors an estimated 38 trillion bacteria spanning at least 1,000 species (Bana and Cabreiro, 2019). The gut microbiota consists of bacteria and commensals, including archaea, viruses, fungi, and protists (Matijašić et al., 2020). Several studies have demonstrated a significant association between specific gut microbiota profiles and T2DM development and progression. For example, *Lactobacillus fermentum* and *Roseburia intestinalis* improve glucose metabolism and insulin sensitivity and suppress proinflammatory cytokines (Lee et al., 2021). Furthermore, the gut microbiota plays an important role in regulating metabolic profiles by producing various metabolites (de Vos et al., 2022). Recent research indicates that serum metabolites, including amino acids, short-chain fatty acids (SCFAs), and various lipid species, play a direct role in bone remodeling (Mann et al., 2024; Yang et al., 2024). Dysregulation of these metabolite profiles in T2DOP, characterized by elevated cholesterol levels and reduced SCFA concentrations, is associated with impaired osteoblast function and increased osteoclastogenesis. For example, SCFAs like acetate and butyrate inhibit osteoclast differentiation through FFAR2 signaling pathways, thereby suppressing bone resorption (Bridgeman et al., 2020; Xie et al., 2025). Conversely, disturbances in glycerophospholipid metabolism contribute to increased skeletal fragility. Targeted therapeutic approaches, including the use of probiotics and metabolic modulators, have demonstrated the potential to restore normal bone metabolism in preclinical models (Qi et al., 2025). Although D + Q modulates gut microbiota and metabolic function in aged mice, the correlation between the gut microbiota and serum metabolites mediated by D + Q in the context of T2DOP remains unclear (Islam et al., 2023; Saccon et al., 2021).

In this study, a T2DOP mouse model was established, and the differences in gut microbiota and serum metabolites with and without D + Q treatment were systematically investigated. Our results indicate that D + Q treatment can modulate the gut microbiota and serum metabolite profiles. Additionally, we identified cell autophagy as a potential target through which D + Q alleviates bone loss. These results indicate that D + Q may be an effective treatment strategy for T2DOP.

## Materials and methods

### Reagents

Dastinib (HY-10181, Purity: 99.83%), Quercetin (HY-18085, Purity: 99.80%), Streptozotocin (HY-13753), PEG400 (HY-Y0873A), and Tween-80 (HY-Y1891) were supplied by MedChemExpress (Shanghai, China). Paraformaldehyde (PFA, BL539A), hematoxylin and eosin staining kit (H&E staining kit, C0105S) was obtained from Beyotime Biotechnology Company (Shanghai, China). Tartrate Resistant Acid Phosphatase (TRAcP) staining kit (BB-4421) was obtained from Bestbio Company (Nanjing, China). Ethylenediaminetetraacetic acid (EDTA, E8008) was purchased from Sigma–Aldrich (Sydney, Australia). Primary antibodies against LC3A/B (D3U4C) was obtained from Cell Signaling Technology (Massachusetts, USA). Primary antibodies against P62 (ab109012) and BECLIN1 (ab302670) were received from Abcam (Cambridge, UK).

### Animals

All animal experiments were conducted in accordance with the guidelines and approval of the Animal Committee of the Laboratory Animal Center of Guangzhou University of Chinese Medicine (no. 20230427002). Male C57BL/6J mice, weighing 20 ± 5 g, obtained from the Laboratory Animal Center of Guangzhou University of Chinese Medicine (Guangzhou, China), were used in this study. The mice were randomly divided into three groups (n = 6 per group): Control (CON), T2DOP model (MOD), and D + Q. Mice in MOD and D + Q groups were adaptively fed for 1 week and then fed on a high-fat diet (HFD) for 4 weeks. All mice, except those in CON group, were intraperitoneally injected with STZ (35 mg/kg) for 3 days to induce T2DM. The D + Q group was then treated with D (50 mg/kg) + Q (50 mg/kg) via intragastric gavage for 7 weeks. After establishing the T2DOP model, body weight and fasting blood glucose (FBG) levels were measured weekly for each group. Mice were subjected to persistent hyperglycemia, and those with blood glucose levels ≥ 12.3 mg/dL were included in MOD and D + Q group.

### Micro-CT

The left femurs of the mice were fixed in 4% PFA at room temperature for 24 h and scanned using the Skyscan 1,172 model (Bruker Corporation, Belgium). The evaluation indices included bone volume over total volume (BV/TV, %), trabecular bone thickness (Tb.Th, mm), trabecular bone separation (Tb.Sp, mm), trabecular bone number (Tb.N, 1/mm), and structure model index (SMI). Before scanning, the parameters were set as follows: 80 kV voltage, 100 μA current, 0.4-degree rotation step, 0.5 mm aluminum filter, and a slice thickness of 5 μm. The index data were analyzed using the CTAn software (Skyscan, Bruker Corporation).

### H&E staining

After fixation, the femurs were decalcified in 10% EDTA for 4 weeks and then embedded in paraffin blocks. The liver tissues were directly embedded in paraffin blocks. All samples were cut into 5 μm sagittal slices and stained using H&E staining solution. A Panoramic Midi Digital Slide Scanner (3D HISTECH Ltd, Hungary) was used for image acquisition. The images were analyzed using SlideViewer 2.6 software (3D HISTECH Ltd, Hungary) and ImageJ software.

### TRAcP staining

The slides were incubated in TRAcP incubation solution at 37°C for 50 min. Osteoclasts were subsequently observed under a microscope. Osteoclasts were considered TRAcP-positive if they turned burgundy. The sections were then stained with Harris Hematoxylin for 3–8 min after rinsing with distilled water. The slides were then soaked in 1% hydrochloric acid and alcohol for a few seconds for differentiation, followed by rinsing with running water. The slices were then treated with 0.6% ammonia to restore their blue color and rinsed again with tap water. Finally, the slides were examined using a panoramic Midi digital biopsy scanner (3D HISTECH Ltd, Hungary), and the figures were analyzed using SlideViewer software (version 2.6; 3D HISTECH Ltd, Hungary) and the ImageJ software.

### Immunohistochemistry (IHC) staining

After antigen retrieval, the sections were incubated with primary antibodies against P21, LC3, P62, Beclin 1, and mTOR, followed by treatment with biotinylated goat anti-rabbit secondary antibodies. Subsequently, the sections were incubated with DAB for 10 min. Image J software was utilized to analyze the images of IHC staining.

### 16S rRNA amplicon sequencing data acquisition

Total genomic DNA was extracted from the samples and used as a template for PCR amplification of bacterial 16S rRNA genes. Amplification was performed using barcoded primers and Ex Taq (Takara). For bacterial diversity analysis, V3–V4 (or V4–V5) variable regions of the 16S rRNA genes were amplified using universal primers 343F (5′-TACGGRAGGCAGCAG-3′) and 798R (5′-AGGGTATCTAATCCT-3′) for V3–V4 regions or 515F (5′-GTGCCAGCMGCCGCGG-3′) and 907R (5′-CCGTCAATTCMTTTRAGTTT-3′) for V4–V5 regions. The quality of the amplicon was assessed using agarose gel electrophoresis. PCR products were purified using AMPure XP beads before undergoing another round of PCR. Sequencing was performed using an Illumina NovaSeq 6,000 with 250 bp paired-end reads (Illumina Inc., San Diego, CA).

### 16S rRNA amplicon sequencing data analysis

Alpha and beta diversity analyses were conducted using QIIME 2 software. Alpha diversity, including the Ace, Chao 1, Shannon, and Simpson indices, was used to estimate the microbial diversity in the samples. To assess beta diversity, the unweighted pair-group method with arithmetic means (UPGMA) was applied using the unweighted UniFrac distance matrix. Significant differences between groups were analyzed using the analysis of variance (ANOVA). The linear discriminant analysis effect size (LEfSe) method was utilized to compare the taxonomic abundance spectra.

### LC-MS/MS data acquisition and processing

Serum metabolic profiles in electrospray ionization (ESI) positive and ESI negative ion modes were analyzed using an ESI source (Thermo Fisher Scientific, Waltham, MA, USA). An ACQUITY UPLC HSS T3 column (100 mm × 2.1 mm, 1.8 μm) was used for both positive and negative modes. The original LC-MS/MS data were processed using Progenesis QI V2.3 software (Non-linear Dynamics, Newcastle, UK).

### Serum metabolomics data analysis

The raw data matrix was imported into the R software for partial least-squares discriminant analysis (PLS-DA) to identify metabolites that differed between the groups. The variable importance in projection (VIP) values obtained from the PLS-DA model were used to rank the overall contribution of each variable to the discrimination between groups. One-way analysis of variance (ANOVA) test was further conducted to verify the significance of the differential metabolites between groups. The threshold values were set as VIP values > 1.0 and *p*-value < 0.05.

### Statistical analysis

All experiments were conducted in triplicate. The data were analyzed using one-way ANOVA and expressed as mean ± standard deviation (SD). Changes in body weight and FBG over time were analyzed using repeated-measures (RM) ANOVA with Group as the between-subjects factor and time as the within-subjects factor, followed by Tukey's HSD where significant effects were detected. The assumptions of RM ANOVA, including normality of residuals (assessed via the Shapiro-Wilk test), sphericity (assessed via Mauchly's test, with Greenhouse-Geisser correction applied where violated), and homogeneity of variances between groups at each time point (assessed via Levene's test), were evaluated. The data met these assumptions sufficiently to proceed with RM ANOVA. Differences with *p* < 0.05 were considered statistically significant. Statistical analyses were performed using GraphPad Prism software (version 9.4.1).

## Results

### D + Q alleviates weight gain and high blood glucose in T2DOP

**Figure 1:**
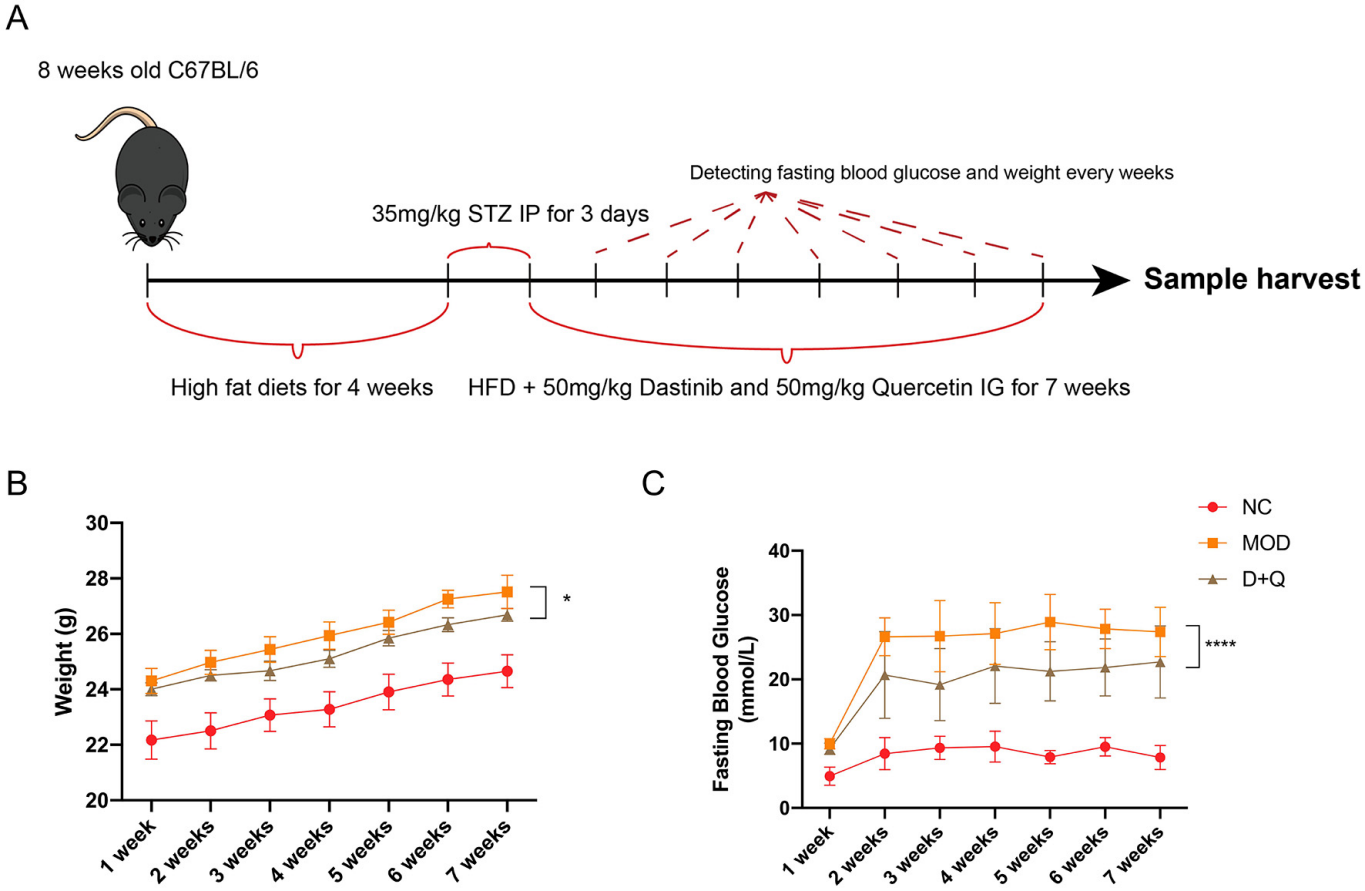
D + Q maintains weight and fasting blood glucose. **(A)** Schematic diagram of the animal experimental design. **(B)** Weight changes during 7 weeks. **(C)** Fasting blood glucose changes during 7 weeks [Data were presented as mean ± SD. *n* = 6 per group. The D + Q group was compared with the MOD group, and significant differences were shown as * (*p* < 0.05) **** (*p* < 0.0001)].

T2DOP mice were successfully induced after 4 weeks of HFD followed by 3 days of STZ intraperitoneal injection (Figure 1A). During the 7-week detection period, T2DOP mice consistently gained weight (Figure 1B). However, D + Q treatment significantly reduced the rate of weight gain (Figure 1B). In T2DOP mice, FBG levels increased rapidly and remained at ~27.43 mmol/L (Figure 1C). After D + Q treatment, FBG levels decreased to ~21.29 mmol/L and reached a plateau (Figure 1C).

### D + Q suppresses bone loss in T2DOP

**Figure 2:**
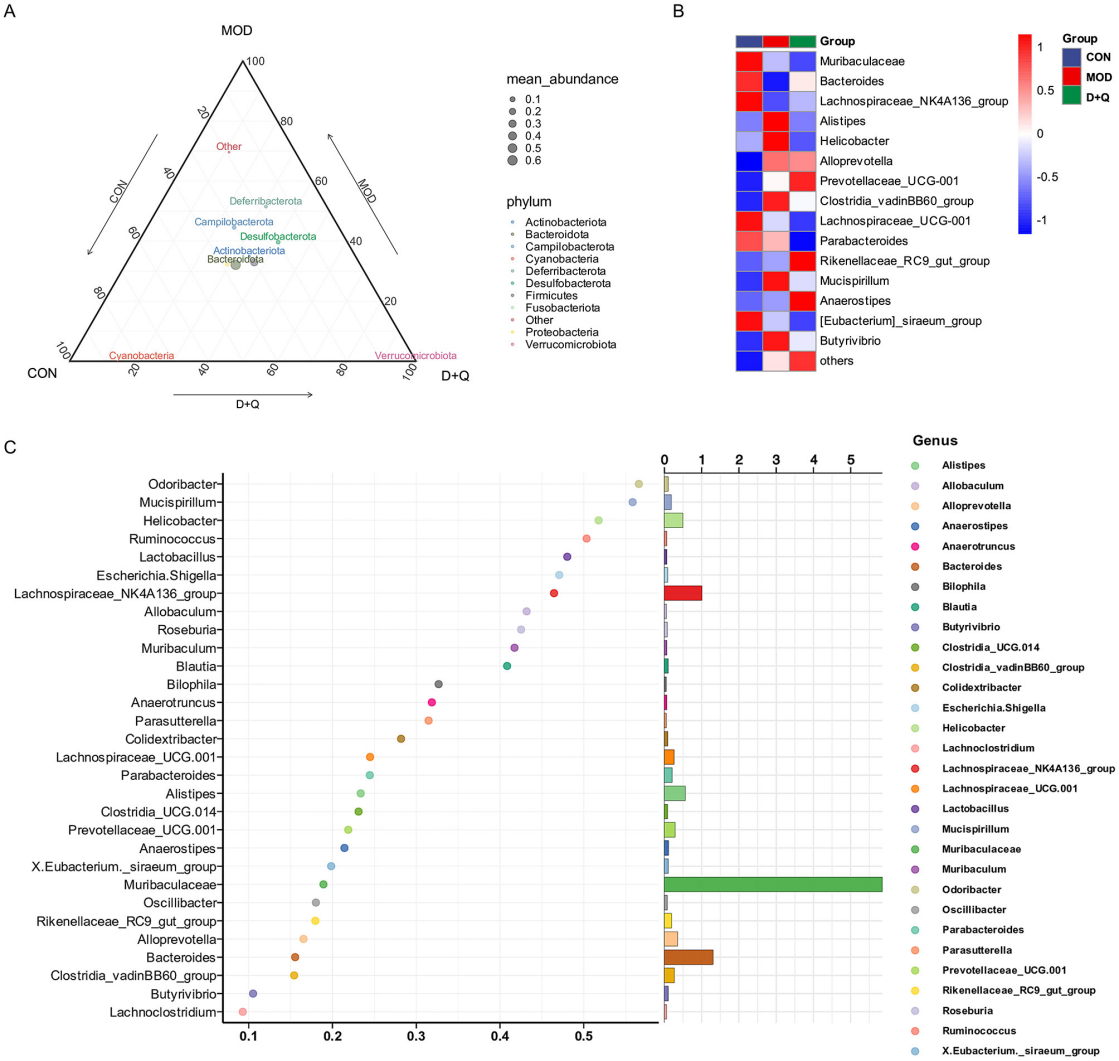
D + Q modulates gut microbiota in T2DOP mice. **(A)** Relative abundance of different phylum among CON, MOD, and D + Q groups. **(B)** Top 15 abundant genera among CON, MOD, and D + Q groups. **(C)** Random forest analysis of the genera.

Three-dimensional reconstruction of the trabecular bone revealed a significant decrease in trabecular bone mass in mice with T2DOP (Figure 2A). After 6 weeks of D + Q treatment, the bone mass was restored (Figure 2A). BV/TV data indicated that D + Q protected the bone volume from the detrimental effects of hyperglycemia (Figure 2B). Moreover, D + Q treatment significantly increased the trabecular bone thickness and decreased trabecular bone number while decreasing the trabecular bone separation (Figures 2C–F). H&E staining revealed an increased number of lipid droplets and a decrease in bone mass in the MOD group, whereas D + Q treatment significantly alleviated bone loss (Figure 2G). In H&E-stained sections of the liver, more lipid droplets were observed in the MOD group, while D + Q treatment significantly reduced the number of lipid droplets (Supplementary Figure 1). No evidence of drug-induced hepatotoxicity was observed across D + Q group. Hepatic architecture remained intact, with no signs of necrosis, inflammation, or degenerative changes (Supplementary Figure 1). The number of TRAcP-positive cells was significantly higher in the MOD group, while D + Q treatment effectively reduced the number of osteoclasts (Figure 2H). To detect the senescence phenotype, the P21 expression level in bone tissue was assessed. The results revealed that D + Q restores p21 expression increased by T2DOP (Figure 2I).

### D + Q modulates the gut microbiome in T2DOP mice

**Figure 3:**
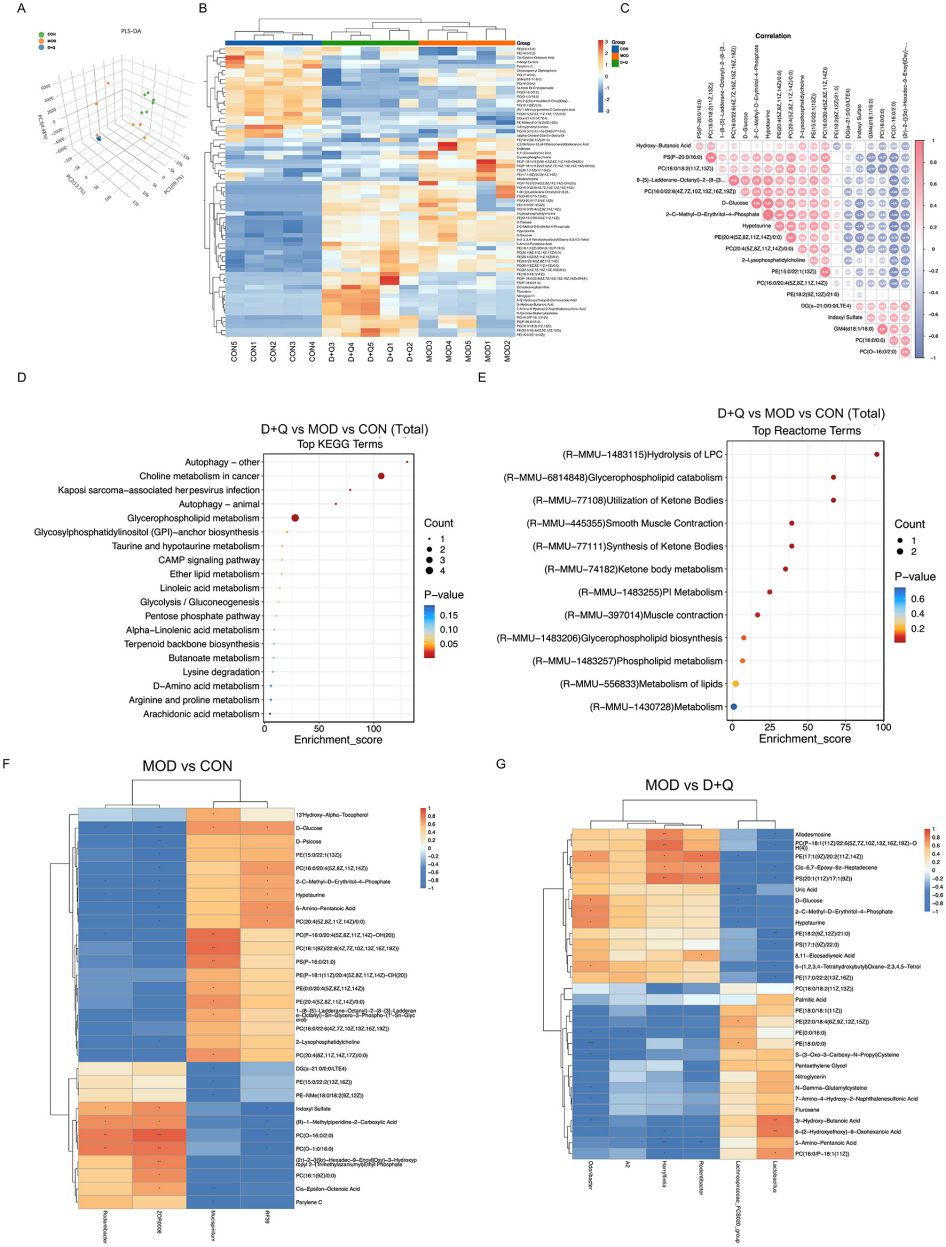
D + Q modulates serum metabolites in T2DOP mice. **(A)** PLS-DA results among CON, MOD, and D + Q groups. **(B)** Heat map of top 50 differential metabolites. **(C)** Top 20 metabolites correlation analysis results. **(D)** KEGG enrichment analysis of metabolic differences among CON, MOD, and D + Q groups. **(E)** Reactome enrichment analysis of metabolic differences among CON, MOD, and D + Q groups **(F)** Heatmap of top 30 significant correlations of MOD group vs. CON group. **(G)** Heatmap of top 30 significant correlations of MOD group vs. D + Q group.

Alpha diversity was analyzed using Ace, Chao 1, Shannon, and Simpson methods. No changes were observed after treatment with D + Q (Supplementary Figures 2A–D). The UPGMA was used to determine beta diversity. The results demonstrated that the gut microbes from the D + Q group were clustered together (Supplementary Figure 2E). The ternary results at the phylum level revealed that *Bacteroidota, Actinobacteriota*, and *Desulfobacterota* exhibited higher relative abundances (Figure 3A). At the genus level, the top 15 abundant species among the three groups were selected based on the results of species annotations, and a columnar cumulative chart of species relative abundance was created to visualize the horizontal community structures across groups with higher relative abundance and their proportions. *Bacteroides* and *Lachnospiraceae_NK4A136_group* were decreased in the MOD group and restored in the D + Q group. *Alistipes, Clostridia_cadinBB60_group, Mucispirillum, Helicobacter*, and *Butyrivibrio* were increased in the MOD group and improved in the D + Q group (Figure 3B). The Random-Forest test revealed that *Mucispirillum, Lactobacillus, Odoribacter, Helicobacter*, and *Ruminococcus* were the top five key microbiomes affected by D + Q (Figure 3C).

### D + Q regulates the serum metabolites in T2DOP mice

**Figure 4:**
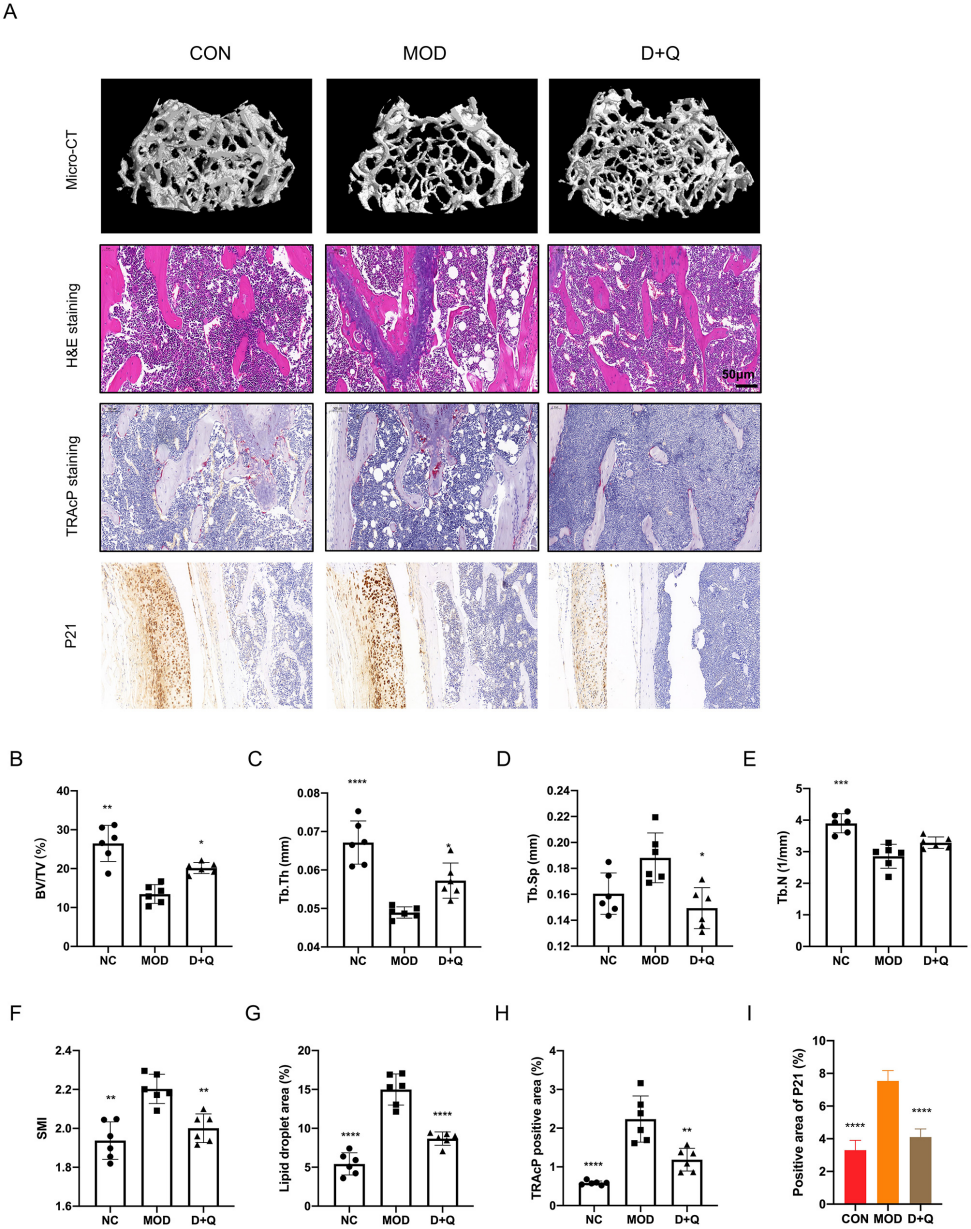
D + Q restores bone mass in T2DOP mice. **(A)** Representative micro-CT 3D remodeling graphic of each group's trabecular bone, H&E staining of femur, TRAcP staining, and IHC staining of femur of each group. **(B–F)** Quantitative presentation of microarchitectural parameters, including BV/TV, Tb.N, Tb.Th, Tb.Sp, SMI. **(G)** Bar plot of lipid droplets fraction area. **(H)** Bar plot of TRAcP positive area. **(I)** IHC of P21 expression level [Data were presented as mean ± SD. *n* = 6 per group. Each group was compared with the MOD group, significant differences were shown as * (*p* < 0.05) ** (*p* < 0.01)].

PLS-DA is a common metabolomic classification method that addresses the limitation of Principal Component Analysis. The PLS-DA score was used to separate the metabolites of CON, MOD, and D + Q groups, indicating processing differences (Figure 4A). The top 12 of D + Q-induced down-regulated metabolites included PE[18:2(9Z, 12Z)/21:0], PE[17:1(9Z)/20:2(11Z, 14Z)], PC [P-18:1(11Z)/22:6(5Z, 7Z, 10Z, 13Z, 16Z, 19Z)-OH(4)], PS[20:1(11Z)/17:1(9Z)], D-Glucose, D-Psicose, Allodesmosine, 2-C-Methyl-D-Erythritol-4-Phosphate, Hypotaurine, 2,2-Dichloro-12-(4-Chlorophenyl)Dodecanoic Acid, 8,11-Eicosadiynoic Acid, and 6-(1,2,3,4-Tetrahydroxybutyl; Figure 4B, Supplementary Figures 3A–L). According to the heatmap of the correlation between serum metabolites, the top four most correlated metabolites were 2-C-Methyl-D-Erythritol-4-Phosphate and Hypotaurine, PS(P-20:0/16:0) and PC[16:0/18:2(11Z,13Z)], D-glucose and 2-C-Methyl-D-Erythritol-4-Phosphate, and D-glucose and Hypotaurine (Figure 4C). KEGG enrichment analysis was used to predict the differential metabolites-related pathways (Figure 4D). The results revealed that D + Q treatment influenced autophagy, glycerophospholipid metabolism, linoleic acid metabolism, and alpha-linoleic acid metabolism. Based on the Reactome terms, differential metabolites modulated by D + Q were primarily associated with LPC hydrolysis, glycerophospholipid catabolism, glycerophospholipid biosynthesis, and lipid metabolism (Figure 4E). To determine whether D + Q-induced metabolite changes were associated with the effects on gut microbiota, we performed Spearman's correlation analyses (Figures 4F–G). The results revealed that *Rodentibacter* was changed in both MOD and D + Q groups. Additionally, *Rodentibacter* was positively associated with serum PE[17:1(9Z)/20:2(11Z, 14Z)], and PS[20:1(11Z)/17:1(9Z)] levels.

### D + Q promotes bone cell autophagy in T2DOP mice

**Figure 5:**
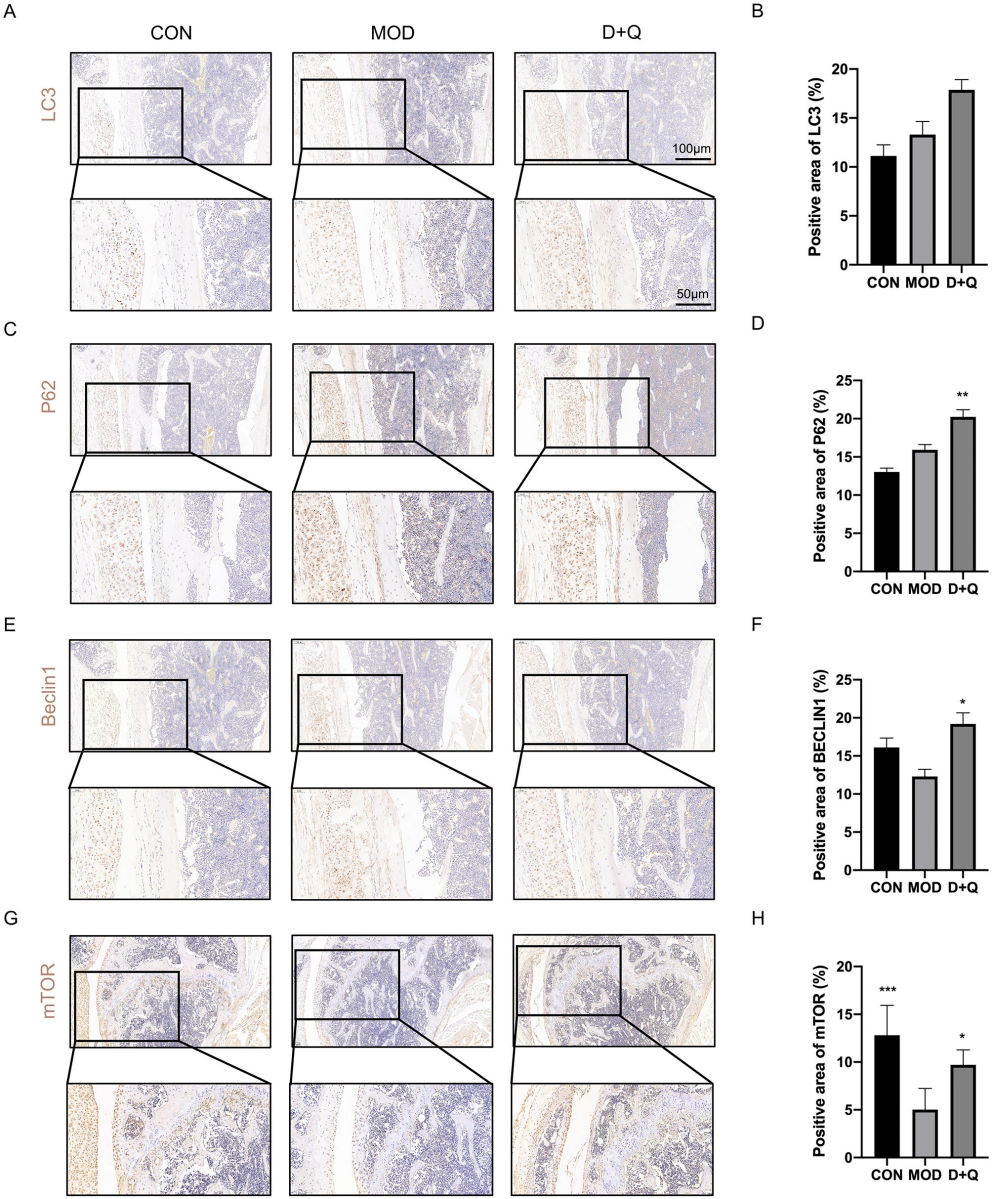
D + Q promotes bone cell autophagy in T2DOP mice. **(A, B)** IHC results of LC3. **(C, D)** IHC results of P62. **(E, F)** IHC results of Beclin1. **(G, H)** IHC results of mTOR [Data were presented as mean ± SD. *n* = 6 per group. Each group was compared with the MOD group, significant differences were shown as * (*p* < 0.05) ** (*p* < 0.01)].

We further performed IHC to determine the effects of D + Q. The cortical bone of the femur demonstrated that D + Q restored the expression levels of LC3, P62, Beclin 1, and mTOR, which were used to quantify autophagy level (Figures 5A–H).

## Discussion

Our results revealed that weight and FBG levels were improved in T2DOP mice. Moreover, D + Q significantly reduced liver droplets, indicating that D + Q restored metabolic disorders in T2DOP mice. Additionally, D + Q treatment resulted in fewer lipid droplets in bone marrow and more trabecular bone. Despite the growing number of patients with T2DOP, there are few available clinical interventions, with hypoglycemic agents having uncertain efficacy (Hofbauer et al., 2022). Recent studies have demonstrated that D + Q could act as a senolytic to decrease senescence cells in T2DM mice (Hickson et al., 2019). D + Q improved FBG and glucose tolerance by removing senescence cells (Islam et al., 2023). We focused on the effect of D + Q on regulating gut microbiome and serum metabolites and discovered that D + Q could play a role in restoring T2DOP-induced bone loss by regulating gut microbiome and serum metabolite profile.

Few studies have revealed that D + Q could ameliorate OP caused by aging and menopause (Wang et al., 2023; Xu et al., 2017). We discovered that D + Q significantly improved trabecular bone mass and microstructure in T2DOP mice. A T2DOP mouse model was developed, which demonstrated that D + Q could decrease lipid droplets in the bone marrow, implying that D + Q could improve osteogenic and adipogenic balance. Peroxisome proliferator-activated receptor-gamma (PPARγ) activation promotes adipogenesis while suppressing osteogenesis via direct transcriptional repression of Runt-related transcription factor 2 (*Run* × *2*; Han et al., 2019). *Run* × *2* inhibits adipogenesis by competing with PPARγ for C/EBPβ binding (Huang et al., 2025). Quercetin reduces the accumulation of lipids in adipocytes, reduces expression of adipogenesis factors like PPARγ (Li et al., 2020). Based on our results, D + Q treatment might rescue bone-fat imbalance by targeting PPARγ-Run × 2. Receptor activator of nuclear factor-kappa B (RANKL) was significantly expressed in senescent osteoblasts that subsequently induced osteoclast differentiation, which could be prevented by administering D + Q (Zhou et al., 2023). Moreover, the same phenotype in T2DOP mice with the TRAcP-positive osteoclasts was decreased by D + Q. Accordingly, D + Q could rescue bone loss by promoting osteogenic activity and directly suppressing osteoclast activity by removing the senescent cells.

Our results revealed that the effect of D + Q was related to regulating the gut microbiome and serum metabolites. The gut microbiota is involved in key physiological processes, including bone metabolism and immune modulation, and gut microbiota imbalance is related to T2DOP (Zhang et al., 2024). The gut–bone axis has recently gained attention because probiotic supplementation increases trabecular bone formation in mice (Xu et al., 2018). We discovered that D + Q could increase the abundance of *Lachnospiraceae_NK4A136_group*. *Lachnospiraceae* could produce SCFAs that interact with the host immune system, reduce proinflammatory responses (Guo et al., 2020). The abundance of *Bacteroides* was increased by D + Q. Additionally, *Bacteroides* was found to produce SCFAs (Tian et al., 2023). Villa et al. demonstrated that *Bacteroides* is positively correlated with bone mass and structure (Villa et al., 2018). Furthermore, D + Q promoted a bloom in *Lactobacillus*, which could suppress bone loss by lowering the chronic inflammation levels (Sui et al., 2022). *Mucispirillum*, which is a proinflammatory bacterium, was decreased by D + Q (Wu et al., 2021).

The gut microbiome could regulate host metabolism and immune function by producing a variety of metabolites, including SCFAs and bile acids (Sanders et al., 2019; Gou et al., 2023). These metabolites could affect bone metabolism. For example, SCFAs could inhibit bone absorption and promote bone formation (Montalvany-Antonucci et al., 2019). Additionally, the gut microbiome can influence bone metabolism by modulating vitamin D and calcium absorption (Lyu et al., 2023). Our results revealed that D + Q changed 66 serum metabolites with increasing levels of amino acids and SCFAs, and reduced cholesterol and triglyceride levels. Amino acids and SCFAs are important nutrients in bone metabolism that can promote osteoblast differentiation and bone formation, and inhibit osteoclast activity (Rizzoli et al., 2021). Based on the enrichment analysis of metabolomics, D + Q could improve metabolic function and promote autophagy, which was consistent with previous studies (Brownlee, 2005; Kirkland and Tchkonia, 2017; Zhu et al., 2024). Additionally, we determined the expression levels of autophagy markers in the femur section and discovered that D + Q could promote bone cell autophagy.

This study had some limitations. First, metabolomic findings were exploratory and require targeted validation. Future studies would functionally interrogate candidate metabolites to establish causal links. Second, we didn't verify the effect of the potential gut microbiome and serum metabolites. Future research could be conducted in further investigation of the specific mechanisms by which D + Q regulates intestinal flora and serum metabolites. Third, the study utilized male mice to control for hormonal variability; however, this limitation restricts the generalizability to female physiology. Future work will incorporate both sexes to evaluate potential sex-specific responses. Exploring whether combining D + Q with other treatments, such as bisphosphonates, can further improve the efficacy.

## Conclusion

Our findings indicate that D + Q treatment significantly alleviated bone loss and improved bone microstructure in mice with T2DOP. This positive effect is attributed to the reduction in weight gain and hyperglycemia, as well as the inhibition of osteoclastogenesis and promotion of osteoblastogenesis. Notably, D + Q induced a favorable shift in the gut microbiome, characterized by a decrease in harmful bacteria like *Muribaculaceae*. Additionally, D + Q modulates serum metabolites, increasing the levels of amino acids and SCFAs that support bone metabolism while reducing cholesterol and triglycerides associated with increased fracture risk. SCFAs producers like *Lachnospiraceae* and *Bacteroides* were increased by D + Q. Thus, we propose that *Lachnospiraceae*-SCFAs and *Bacteroides*-SCFAs serve as high-priority target axes for such future investigations. Future research should focus on elucidating the precise mechanisms underlying the effects of D + Q on bone health.

## Data Availability

The original contributions presented in the study are publicly available. This data can be found here: https://www.ncbi.nlm.nih.gov/, accession OMIX011491.
