# Supplementary material for: Dasatinib and Quercetin alleviate type 2 diabetic osteoporosis by regulating serum metabolite and gut microbiome
Source: Front Microbiol. 2025 Sep 3;16:1631082. doi: 10.3389/fmicb.2025.1631082 (PMC12442493; doi:10.3389/fmicb.2025.1631082)
Supplement: Supplementary file 1 [file Supplementary_file_1.docx]

Supplementary figures


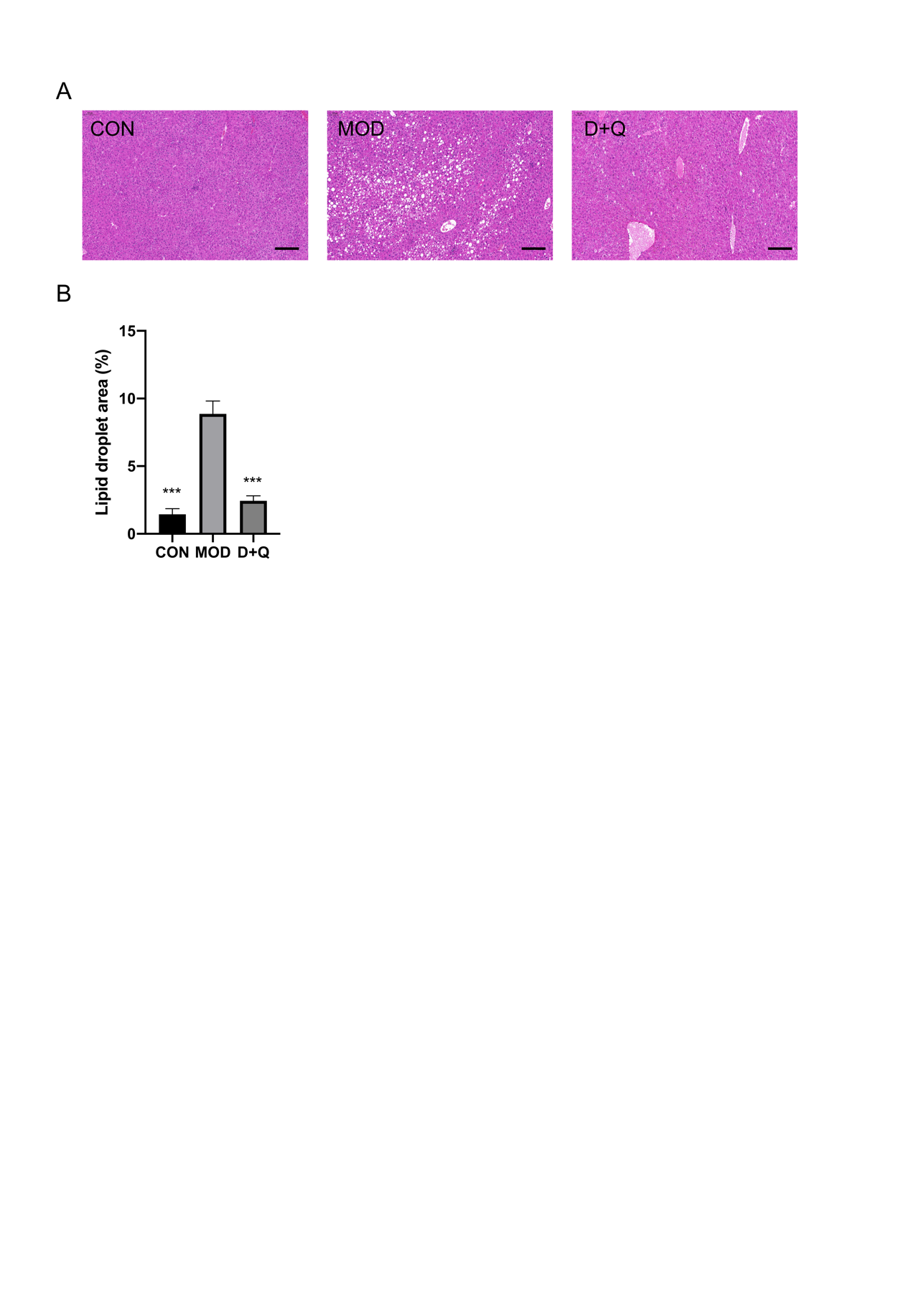


**Supplementary figure 1. (A) Representative H&E staining of liver of each group. (B) Bar plot of lipid droplets fraction area.** **(Data were presented as mean ± SD. n = 6 per group. Significant differences were shown as *** (p < 0.001).)**


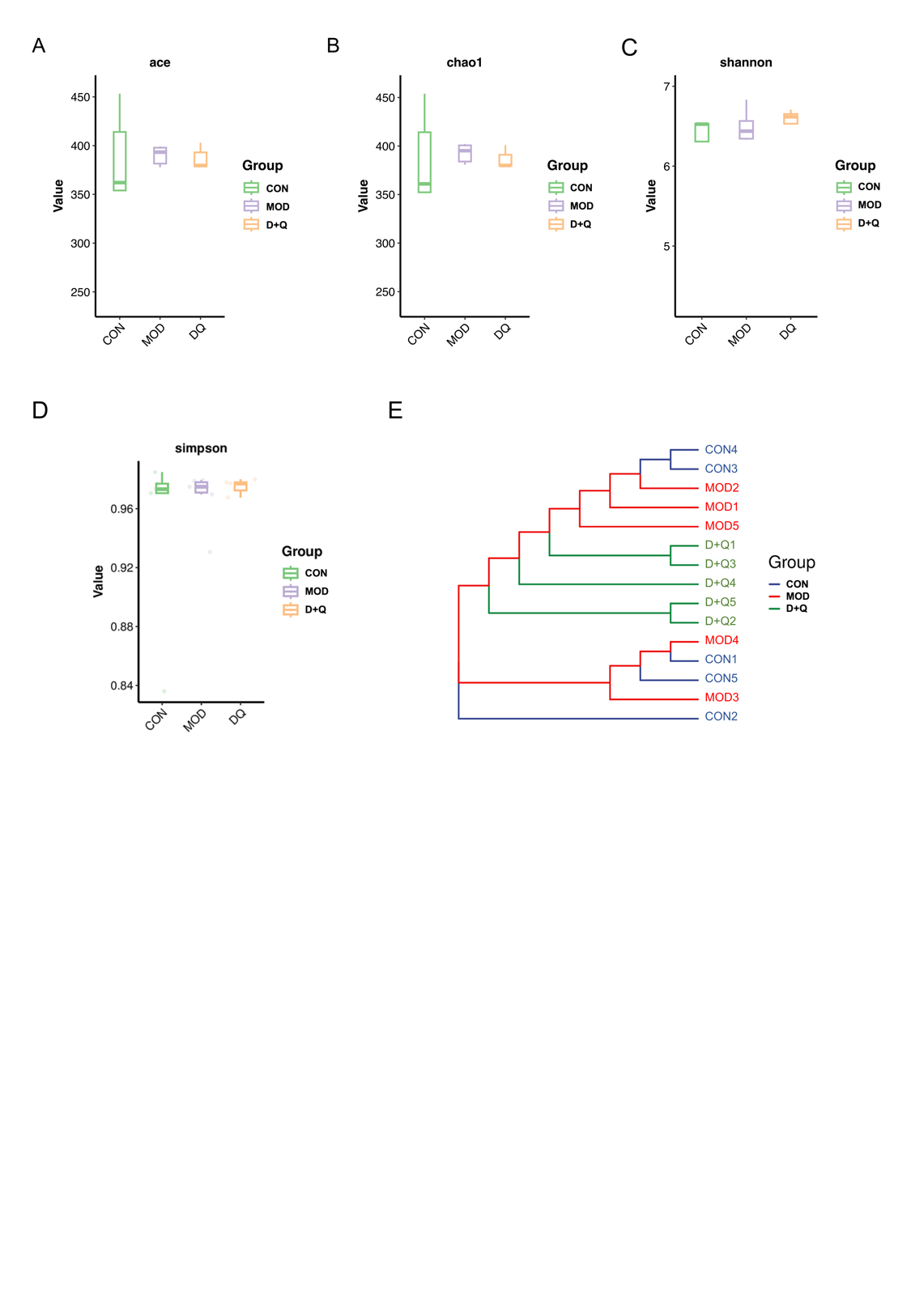


**Supplementary figure 2. (A-D) Alpha diversity analyses of ace, chao1, Shannon, and Simpson.** **(E) Beta diversity of the UPGMA.**

**Supplementary figure 3. (A-L) Bar plot of top 12 of the down-regulated metabolites.**
